# Fear of Violence among Colombian Women Is Associated with Reduced Preferences for High-BMI Men

**DOI:** 10.1007/s12110-019-09350-8

**Published:** 2019-07-31

**Authors:** Martha Lucia Borras-Guevara, Carlota Batres, David I. Perrett

**Affiliations:** Perception Lab, School of Psychology and Neuroscience, University of St Andrews, St Mary’s Quad, St Andrews, Fife, Scotland KY16 9JP UK

**Keywords:** Violence, BMI, Mate choice, Intrasexual competition

## Abstract

Recent studies reveal that violence significantly contributes to explaining individual’s facial preferences. Women who feel at higher risk of violence prefer less-masculine male faces. Given the importance of violence, we explore its influence on people’s preferences for a different physical trait. Masculinity correlates positively with male strength and weight or body mass index (BMI). In fact, masculinity and BMI tend to load on the same component of trait perception. Therefore we predicted that individuals’ perceptions of danger from violence will relate to preferences for facial cues to low-BMI. In two studies in Colombia, men and women from Bogota, Medellin, and surrounding communities were shown pairs of faces transformed to epitomize the shape correlates of men with high or low-BMI. The images were of European, Salvadoran, or Colombian men. Participants were asked to choose the face they considered most attractive. Subsequently, participants answered a survey about their health (e.g., frequency of illnesses the past year), media access (e.g., frequency of Internet use), education level (e.g., graduating from high school), and experiences/perceptions of violence in study 1 and about specific types of violence (public and domestic) in study 2. Results from both studies showed that women who experienced/perceived higher levels of violence preferred faces of low-BMI Salvadoran men. Preferences for low-BMI facial cues were significantly explained by violence (public or domestic), even after controlling for all other variables (including age, education, health, and media access). These results may reflect women’s strategy to avoid male partners capable of inflicting harm.

**Electronic supplementary material:**

The online version of this article (10.1007/s12110-019-09350-8) contains supplementary material, which is available to authorized users.

## Theoretical Background

Violence has been suggested to have influenced male and female psychology in ancestral times. As humans evolved, conflict within and between groups, homicide, and resource theft were prevalent and could have affected female and male psychology (Biocca 1971; Gat 1999, 2000a, b; LeBlanc 2003; Morgan 1980; Puts 2010). Women’s mating psychology could have been greatly affected since women are generally smaller, shorter, and have a different muscle/fat ratio (body composition) compared to men (Frayer and Wolpoff 1985), which in turn made them vulnerable to being hurt in violent contexts (Buss 1994; Buss and Schmitt 1993; Ellis 1992; Symons 1979). Likewise, men’s psychology could have been influenced in scenarios where intrasexual competition was high and the ability to recognize the strongest potential rivals or allies was beneficial (Borráz-León et al. 2014).

Women’s preferences for formidable partners (who are more masculine, stronger, and larger) have been explained in terms of both benefits and costs to women (Borras-Guevara et al. 2017a, b; Snyder et al. 2011). Most studies have focused on the benefits. For example, Snyder and colleagues (Snyder et al. 2011) found that women prefer partners who are more formidable (men described as dominant, tough guy, powerful, strong, could win a fight) when they feel at higher risk from crime. Likewise, Ryder et al. (2016) found that in public places where women feel there is a higher risk of crime, more dominant and formidable males are preferred. Both Snyder et al. and Ryder et al. argue that these results support the idea that women can recognize traits in men associated with being a better protector in dangerous environments. Additionally, Little et al. (2011) found that female participants primed with images of fights between men preferred more masculine male faces. Collectively this research suggests that more formidable partners are better prepared for intrasexual competition and could be more attractive to women looking for protection.

Two types of evidence have been found in support of masculine, formidable-looking men being advantageous for intrasexual competition, and hence protection. One type of evidence relates to men’s actual physical attributes and the other, to perceived attributes and/or descriptions of traits. In reference to men’s physique, Fink et al. (2007) studied the relationship between men’s actual strength and perceived facial masculinity. Women rated the faces of stronger men as more masculine/dominant than weaker men. Additionally, Windhager and colleagues (Windhager et al. 2011) found that men’s upper body strength was positively related to facial features common to masculinity and dominance. Together, Fink et al.’s and Windhager et al.’s results contribute to evidence supporting the claim that masculinity reflects men’s fighting capabilities. In terms of verbally listed characteristics, women cared more about being protected than about any other trait when choosing male friends (Bleske-Rechek and Buss 2001). Further, Greiling and Buss (2000) found that one of women’s most important criteria in choosing a short-term partner was how much protection they could provide.

Recognizing that women face a trade-off when choosing men who can both protect and hurt them is crucial if we want to understand women’s preferences. Choosing male partners who are better suited for conflict (formidable, masculine, stronger, aggressive) (Archer and Thanzami 2007, 2009; Sell et al. 2009) implies that women put themselves at higher risk of being hurt by these same men. Indeed, there is evidence that women’s masculinity preferences are contingent on the type of violence women experience. For example, women shown images of men hitting women felt angry and disgusted, and their masculinity preferences were lowered (Li et al. 2014). Additionally, Colombian women preferred less-masculine male faces when they had higher perceptions of men being dangerous to their children (Borras-Guevara et al. 2017a) and when they were concerned about domestic violence (Borras-Guevara et al. 2017b).

Besides masculinity, another physical characteristic of men that is relevant to formidability is body mass index (BMI). BMI is an indirect indicator of body fat (and muscle) based on weight scaled by height (kg/m^2^). Holzleitner et al. (2014) found that perceptions of masculinity were affected by facial features related to weight. Women perceptions associated more-masculine men’s faces to heavier men. Additionally, Phalane et al. (2017) found that facial perceptions of masculinity and adiposity of men’s faces loaded highly (0.90 and 0.78, respectively) on the same principal component. Furthermore, perceptions of strength from men’s faces were highly related (27% of the variance in the data explained) to facial morphological cues of BMI, so men who are perceived as stronger have faces that look heavier (Holzleitner and Perrett 2016). Strength contributes to how dangerous an individual can be. If women are sensitive to cues to men’s fighting capabilities, and are avoiding these when there is a risk of violence, then we predict that these conditions will promote women’s preferences for male faces showing cues to low-BMI.

In terms of men’s psychology, previous studies have shown that men’s masculinity preferences are congruent to those of women, suggesting that men can recognize potential male rivals who present danger to their partners and resources (Batres and Perrett 2014; Borras-Guevara et al. 2017a; Borráz-León et al. 2014; Perrett et al. 1998; Scott et al. 2013; Swami and Tovée 2005). In fact, Colombian men’s preferences parallel women’s preferences for less-masculine male faces when men are perceived to be a danger to children (Borras-Guevara et al. 2017a). Additionally, men’s preferences for high masculinity in other men may reflect a need for strong potential allies in the face of conflict (Borráz-León et al. 2014). Men’s BMI preferences for male faces may face the same trade-off as women’s. If more-masculine and heavier men are stronger, preferring an ally who is heavy (high BMI), and strong, may constitute a high risk in the context of intragroup conflict but be an asset for intergroup disputes.

In the current study, we looked at the link between BMI preferences for male faces and perceptions of danger from violence (public and domestic) in a Colombian population. As noted above, masculinity and BMI are both positively correlated with actual strength in men (Fink et al. 2007; Windhager et al. 2011; Wolff and Puts 2010), and people prefer less-masculine male faces in different violent contexts (Borras-Guevara et al. 2017a, b). Previous studies in Colombia have shown that women who fear public and domestic violence have lower masculinity preferences for male faces who resemble participants most closely. Although men’s masculinity preferences were also low if they feared violence, these effects were not significant (Borras-Guevara et al. 2017a). Therefore we predicted that preferences for facial cues of BMI would be lower in environments where people have fears about different types of violence. More specifically, we predicted that individuals who worry and have had more experiences of domestic and public violence should display lower preferences for facial cues of BMI in men. Additionally, we predict that violence effects will be more evident for the most ethnically relevant stimuli. In the case of women, preferences may reflect mate choice. For men, preferences may reflect awareness for what women like or the pursuit of safe allies. Since previous studies have shown that BMI preferences are affected by social indicators other than violence, we control for their effects here. Hence, our model included BMI preferences as the dependent variable and education, media access (TV and Internet), access to health services, illnesses, age, and parenthood as covariates. These factors may contribute to differences in environmental harshness that are associated with overall population differences in preferences for higher BMI (e.g., Swami and Tovée 2005).

## Field Site

Two major cities in Colombia, Bogota and Medellin, and surrounding suburban areas were the epicenters of our data collection. This country was chosen as our field site because of its high levels of homicide (United Nations Global Study on Homicide 2013), which increases our chances of finding effects of violence on preferences for facial correlates of BMI. Additionally, since social indicators vary between cities and small towns, we collected data from urban and suburban populations that experience a variety of levels of privation (access to media, health, and education) to increase the power of our design and ensure that our sample is as representative of the Colombian population as possible.

The majority of studies on preferences for facial cues of BMI have been done online, with participants being mostly students or individuals from industrialized countries (e.g., the UK and the US). By contrast, this study involved participants from different backgrounds, ranging from educated individuals from an urban environment to participants with very little access to economic development and education. Furthermore, since online samples have been found not to be representative of the population as a whole in developing countries, all our participants were personally interviewed (Batres and Perrett 2014). The data are provided as Electronic Supplementary Material.

## Study 1

### Methods

#### Participants’ Recruitment

Participant recruitment involved close collaboration with locals and community leaders, who gave assurances and vouched for the objectives of the research to other people in the community and hence helped in getting people to participate in the current study. In order to avoid sample bias, several leaders were approached in different parts of the city and rural areas where data collection occurred.

#### Participants

Colombians living in Bogota or in small towns (population < 10,000) located at least two hours from Bogota were selected as the study population. One hundred and sixty-one participants (80 women and 81 men) were interviewed in Bogota and nearby suburban areas. Participants recruited outside of Bogota were mostly from the states of Magdalena (56%) and Cundinamarca (26%), with the remainder (18%) being from the states of Bolivar, Meta, or Tolima. The minimum age for recruited individuals was 17, but there was no maximum age for participation in this study. Since we were interested in participants who were still in their reproductive phase we considered here only participants less than 41 years old. The effects presented here were also found, however, when older people were included in our analysis. After taking into account this age restriction, we were left with 132 participants, of whom 63 were men (mean age ± SD = 29.98 ± 5.3) and 69 were women (mean age ± SD = 27.89 ± 6.2). For the analyzed sample, 92 participants were in a committed relationship, while only 40 were single. In terms of sexual orientation (scale 1–7, 1 = completely homosexual, 4 = bisexual, 7 = completely heterosexual), only one participant disclosed a rating below 5. Only responses from participants who self-reported as heterosexuals were included.

#### Stimuli

**Fig. 1:**
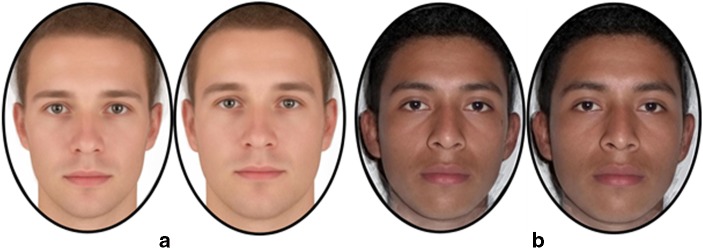
Average of the five pairs of European (**a**) and Salvadoran (**b**) male facial stimuli (shown in the field). The left of each pair corresponds to the average face showing cues of low-BMI and the right shows cues of high BMI

Participants were shown facial photographs of European and Salvadoran men. Colombia and El Salvador are both countries of Latin American Hispanic descent; we included Salvadoran face pictures to vary the familiarity to the facial stimuli. These photographs were all taken under the same camera and lighting conditions, showing no expression and no adornments (either make-up or jewelry) and facing forward. The European facial images were chosen from an online library (3DSK), whereas the Salvadoran images were previously collected in the field (Batres and Perrett 2014). All images were aligned to a standard interpupillary distance and were delineated using 189 point throughout the entire face space (e.g., 25 points delineating the nose). The measured weight and height of the photographed individuals were used to calculate their BMI. Prototypes for high and low correlates of BMI were made by averaging male faces belonging to individuals with the 10 highest (European M  =  26.47 kg/m^2^, SD  =  3.27; M_age_  =  24.80 years, SD  =  3.77; Salvadoran M  =  27.27 kg/m^2^, SD  =  1.96; M_age_  =  22.00 years, SD  =  1.56) and 10 lowest (European M  =  22.19 kg/m^2^, SD  =  2.52; M_age_  =  25.10 years, SD  =  3.96; Salvadoran M  =  20.91 kg/m^2^, SD  =  2.22; M_age_  =  21.20 years, SD  =  1.87) BMI. Five individual composites were made by averaging three unique male faces for each composite. This was done for each ethnicity. We used composite images because it avoids disclosure of an individual’s identity (since the blend of 3 individuals obscures the identity of all 3 faces). This is particularly important when collecting data in places where the stimuli might be recognized. Using the prototypes, ±50% shape difference transforms were made for each composite, keeping color and texture constant, resulting in 10 pairs of faces, 5 European and 5 Salvadoran. Each pair of faces consisted of one face with cues for men’s low-BMI and the other for men’s high BMI (Fig. 1). All procedures related to the making of composites and transforms were made with Psychomorph (http://users.aber.ac.uk/bpt/jpsychomorph/).

#### Procedure

Participants were interviewed individually. They performed an attractiveness forced-choice task and then answered a 57-question survey. In the forced-choice task, participants were shown pairs of male faces representing high and low-BMI. For each pair, the position (left or right) of the faces was alternated. Participants had to choose the face they considered to be the most attractive and were given no time limit. The subsequent survey consisted of five sections with questions relating to demographics, indicators of health, education, media access, and experiences/perceptions of violence (Borras-Guevara et al. 2017a). Demographic questions included participant’s age, sex, number of children, and relationship status. Health questions included access to hospital, potable water, how frequently participants were ill during their childhood, and how many times they had been ill over the past year. Questions relating to education included attendance at high school, graduating from high school, and attendance at university. Questions about media access included ownership of a TV at home and how frequently the Internet was used. Experiences and perceptions of violence were documented by asking participants how much danger from violence they perceived at home, in the city, and in the country (Colombia); as well as how many times had participants been robbed or attacked in the past year and how much they thought that men were a danger to their children. Once participants finished answering all the questions, they were debriefed and paid £5 (20,000 Colombian pesos) for their time.

### Statistical Analysis

A repeated measures ANCOVA was run because our experimental design was particularly suited for this type of statistical analysis. Our design was simple, with no missing data; repeated measures were categorical and balanced (three ethnicities with five pairs of faces per ethnicity), and residuals were normal. At first glance, our dependent variable may seem binary; however, for each ethnicity we use the percentage of high-BMI faces chosen in the five forced-choice trials (high vs. low BMI).

#### Dependent Variable

The number of times that participants chose the high-BMI face as most attractive was computed as a percentage. This resulted in two scores: percentage preference for facial cues associated with high-BMI Salvadoran males and percentage preference for facial cues associated with high-BMI European males.

#### Independent Variables

When possible a principal component analysis (PCA) was run and factors were extracted to reduce the number of variables for each section in the survey (education, health, violence perceptions, access to media, demography). The suitability of each factor analysis was assessed by the Keyser-Meyer-Olkin (KMO) test. This test measures the proportion of variance in the data explained by the model. Values range from 0 to 1, with numbers <0.7 indicating suitability for this type of analysis. All solutions were unrotated and based on eigenvalues greater than 1. The loadings for all factors are shown in Table 1. For each PCA, scores were saved as new variables/factors.

**Table 1:** Factor names and loadings: factors extracted for each indicator (caps) with all loadings. Numbers in **bold** are >0.44 or < −0.44). Factors are referred to hereafter by their name alone (e.g., public and domestic violence)

Indicators	Factor names and loadings	
VIOLENCE	Public violence	Domestic violence
Home danger	**0.55**	−0.29
City/town danger	**0.83**	0.10
Country danger	**0.75**	0.44
Robbed frequency	**0.56**	−0.41
Men danger to children	−0.28	**0.85**
HEALTH	Health access	Illnesses
Drinking water	−**0.55**	−0.38
Born in a hospital	**0.71**	−0.05
Access to hospital	**0.80**	0.23
Average illnesses	−0.34	**0.64**
Childhood illnesses	−0.18	**0.79**
EDUCATION	Education	
Attended high school	**0.75**	
Graduated high school	**0.85**	
Attended university	**0.70**	

From the five questions relating to violence perceptions and experiences (perceptions of danger in the home, in the city, in the country, frequency of robberies experienced in the last year, and how much did participants agree with the statement “men are dangerous to their children”), two factors were extracted, explaining 37.2% and 23.4%, respectively, of the variance in the data. The first factor was mostly loaded with questions related to public violence. The second factor loaded mostly on the question of how much participants agreed that men were dangerous to their children. For questions relating to education (attending high school, graduating from high school, and attending university), only one factor was extracted, explaining 58.6% of the variance. In reference to the five questions asked about health (drinking water, hospital access, being born in a hospital, frequency of illnesses during childhood, and average illnesses during the last year), two factors were extracted. The first factor explained 31.9% of the variance and heavily loaded on questions relating to access to health services (access to drinking water, being born in a hospital, and access to a hospital). The second factor related to illnesses and explained 24.7% of the variance. Since all participants reported having a television, frequency of Internet use was the only question used to represent media access.

### Results

#### Preferences for High-BMI Males

A repeated-measures, within-subjects ANCOVA was conducted with preferences for facial cues of high-BMI males as the dependent variable, ethnicity (European vs. Salvadoran) as a within-subject factor, and participant’s sex and having children as between-subject factors. Covariates in this model were media access (Internet use), participant’s age, and factors relating to illnesses, health access, and public and domestic violence. BMI preferences’ residuals were normally distributed (skewness and kurtosis between 0.74 and − 0.74). Including relationships status in the model did not contribute to explaining variance in the preferences for cues to BMI in male faces; in fact, since all significant effects and their directions were the same when this variable was included in the model, it was excluded from any subsequent statistical analysis.

The only significant main effect found in this model was having children (F_1,117_ = 6.08, *p* = 0.015, η^2^ = 0.049, β = −8.79, CI[−45.94, −2.98). Colombians who had children had lower preferences for facial cues associated with high-BMI males. There were no other significant main effects: participant’s sex (F_1,117_ = 0.552, *p* = 0.47, η^2^ = 0.004, β = −2.56, CI[−11.51, 6.39]), media access (F_1,117_ = 0.16, *p* = 0.69, η^2^ < 0.001, β = 0.95, CI[−3.76, 5.67]), age (F_1,117_ = 0.36, *p* = 0.55, η^2^ = 0.003, β = 0.17, CI[−0.40, 0.76]), domestic violence (F_1,117_ = 0.12, *p* = 0.73, η^2^ = 0.001, β = −0.61, CI[−4.12, 2.90]), public violence (F_1,117_ = 2.11, *p* = 0.15, η^2^ = 0.018, β = −2.47, CI[−5.83, 0.89]), education (F_1,117_ = 1.6, *p* = 0.21, η^2^ = 0.014, β = 2.41, CI[−1.36, 6.19]), health access (F_1,117_ = 1.95, *p* = 0.17, η^2^ < 0.016, β = 2.40, CI[−1.01, 5.81]) and illnesses (F_1,117_ = 0.00, *p* = 0.99, η^2^ < 0.001, β = −0.02, CI[−3.48, 3.48]). The interaction between participant’s sex and having children was not significant either (F_1,117_ = 2.28, *p* = 0.13, η^2^ = 0.019).

**Fig. 2:**
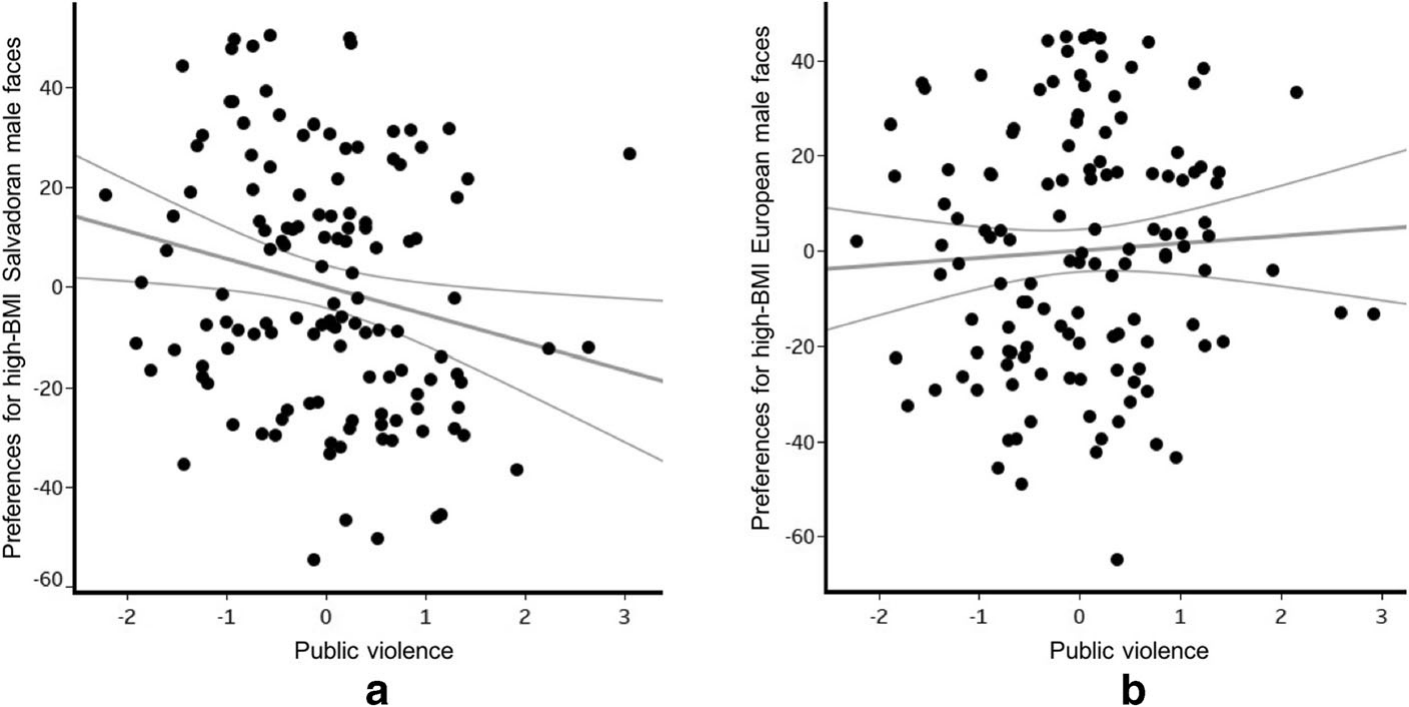
Effect of the public violence factor on preferences for facial cues to high BMI (**a**) Salvadoran and (**b**) European males. Each black dot represents an individual; gray lines indicate the 95% confidence interval. (Residual public violence plotted against high BMI preferences unstandardized residuals, controlling for all other covariates)

Face stimulus ethnicity significantly interacted with the public violence factor (F_1,117_ = 5.5, *p* = 0.021, η^2^ = 0.045). When participants had higher perceptions/experiences of public violence, their preferences for facial cues of high-BMI were lower for Salvadoran faces but unchanged for European faces (Fig. 2).

Additionally, there was a significant three-way interaction between stimulus ethnicity, participant’s sex, and having children (F_1,117_ = 10.43, *p* = 0.02, η^2^ = 0.082). None of the other covariates in the model, namely participant’s sex (F_1,117_ = 0.17 *p* = 0.68, η^2^ = 0.001), media access (F_1,117_ = 1.10, *p* = 0.29, η^2^ = 0.009), age (F_1,117_ = 0.009, *p* = 0.92, η^2^ < 0.001), domestic violence (F_1,117_ = 0.58, *p* = 0.45, η^2^ = 0.005), health access (F_1,117_ = 0.46, *p* = 0.50, η^2^ = 0.004), illnesses (F_1,117_ = 2.91, *p* = 0.09, η^2^ = 0.024), education (F_1,117_ = 1.21, *p* = 0.27, η^2^ = 0.001), and having children (F_1,117_ = 2.32, *p* = 0.13, η^2^ = 0.019), significantly interacted with the ethnicity of the face shown to participants.

Because there were significant interactions between the ethnicity of the facial stimulus used and the public violence factor, and between facial stimuli, having children, and participant’s sex, separate analyses were run for each sex and for each face ethnicity.

#### Men’s Preferences for High-BMI European Males

Men’s age (F_1,58_ = 7.78, *p* = 0.033, η^2^ = 0.087, β = −1.66, CI[−3.19, −0.14]) had a significant effect on preferences for high-BMI European males. Older participants showed a lower preference for facial correlates of high-BMI European males. None of the other covariates (health access, illnesses, education, media access, public violence, or domestic violence) had a significant effect on these preferences, and they hardly contributed to any variation in this model: health access (F_1,58_ = 0.12, *p* = 0.73, η^2^ = 0.002, β = 1.05, CI[−4.99, 7.08]), illnesses (F_1,58_ = 0.34, *p* = 0.56, η^2^ = 0.007, β = −2.47, CI[−10.91, 5.98]), education (F_1,58_ = 0.81, *p* = 0.37, η^2^ = 0.016, β = −3.37, CI[−10.87, 4.13]), media access (F_1,58_ = 1.38, *p* = 0.24, η^2^ = 0.027, β = 5.76, CI[−4.07, 15.58]), public violence (F_1,58_ = 0.001, *p* = 0.98, η^2^ < 0.001, β = 0.09, CI[−7.71, 7.89]), and domestic violence (F_1,58_ = 0.42, *p* = 0.52, η^2^ = 0.008, β = −2.47, CI[−10.14, 5.20]).

#### Men’s Preferences for High-BMI Salvadoran Males

Men’s preferences for facial cues of high-BMI Salvadoran males were significantly affected by having children (F_1,58_ = 3.76, *p* < 0.001, η^2^ = 0.28, β = −30.73, CI[−44.71, −16.76]). Men with children had a higher preference for Salvadoran male faces showing correlates of high-BMI. Neither participant’s age (F_1,58_ = 0.09, *p* = 0.77, η^2^ = 0.002, β = −0.19, CI[−1.52, 1.13]) nor any of the other factors had a significant effect on this type of preferences: media access (F_1,58_ = 0.33, *p* = 0.57, η^2^ = 0.007, β = −2.43, CI[−10.96, 6.09]), education (F_1,58_ = 1.4, *p* = 0.24, η^2^ = 0.028, β = 3.85, CI[−2.65, 10.37]), domestic violence (F_1,58_ = 0.24, *p* = 0.63, η^2^ = 0.005, β = 1.62, CI[−5.03, 8.27]), public violence (F_1,58_ = 2.22, *p* = 0.14, η^2^ = 0.043, β = −5.02, CI[−11.79, 1.74]), illnesses (F_1,58_ = 2.86, *p* = 0.09, η^2^ = 0.054, β = 6.16, CI[−1.16, 13.49]) and health access (F_1,58_ = 0.6, *p* = 0.44, η^2^ = 0.012, β = 2.02, CI[−3.21, 7.25]).

#### Women’s Preferences for High-BMI European Males

Neither participant’s access to media (F_1,59_ = 0.43, *p* = 0.51, η^2^ = 0.057, β = 3.60, CI[−7.36, 14.56]) or any of the covariates (health access, illnesses, education, public violence, domestic violence or having children) had a significant effect on preferences for facial cues to high-BMI European males and hardly contributed to any variation in these preferences: health access (F_1,59_ = 0.32, *p* = 0.57, η^2^ = 0.005, β = 4.93, CI[−12.55, 22.1]), illnesses (F_1,59_ = 0.65, *p* = 0.42, η^2^ = 0.005, β = −2.77, CI[−11.37, 5.82]), education (F_1,59_ = 0.25, *p* = 0.62, η^2^ = 0.004, β = 2.28, CI[−6.82, 11.37]), public violence (F_1,59_ = 1.05, *p* = 0.31, η^2^ = 0.017, β = 3.44, CI[−3.26, 10.13]), domestic violence (F_1,59_ = 1.14, *p* = 0.29, η^2^ = 0.019, β = −4.00, CI[−11.52, 3.50]) and having children (F_1,59_ = 2.43, *p* = 0.12, η^2^ = 0.039, β = −13.25, CI[−30.24, 3.75]). Women’s age, however, did show a trend (F_1,59_ = 3.62, *p* = 0.062, η^2^ = 0.057, β = 1.01, CI[−0.05, 2.08]). Older women had higher facial preferences for high-BMI European males.

#### Women’s Preferences for High-BMI Salvadoran Males

**Fig. 3:**
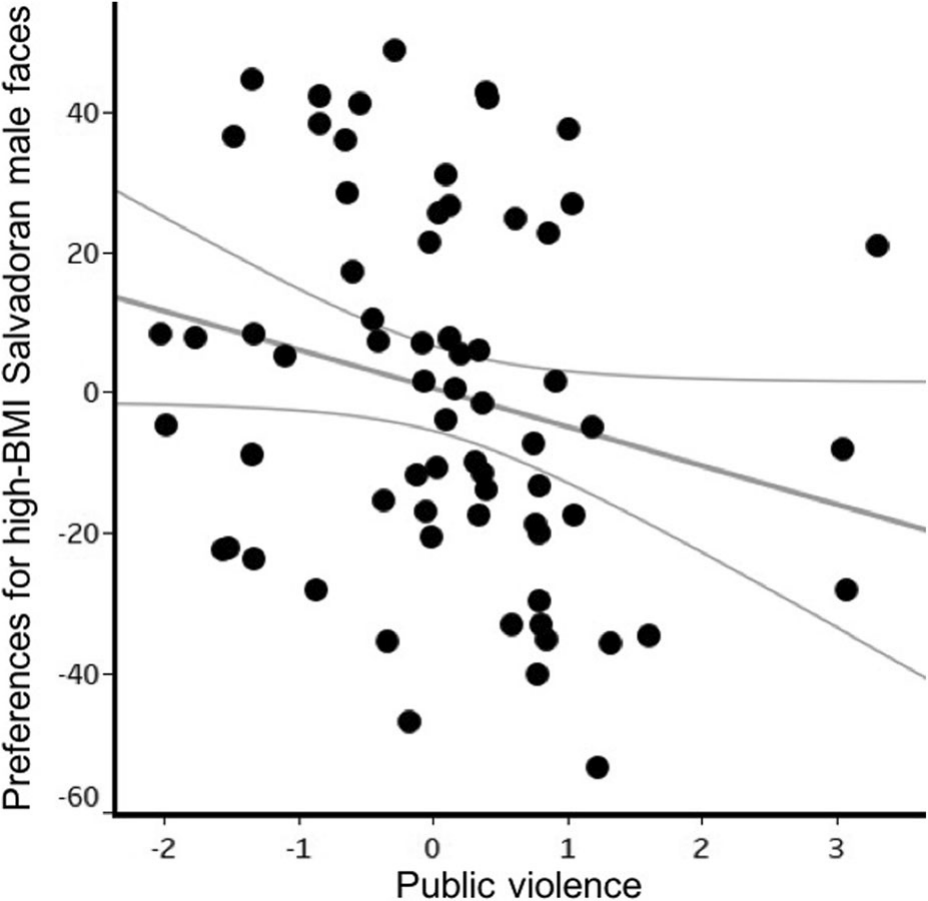
Effect of the public violence factor on women’s preferences for facial cues to high BMI Salvadoran males. Each black dot denotes a participant and grey lines show a 95% confidence interval. Unstandardized residuals of the public violence factor plotted against the unstandardized residuals of preferences for high BMI, controlling for age, access to media, domestic violence, access to health, illnesses, education, and having children

Women’s preferences for facial cues to high-BMI Salvadoran males were significantly affected by public violence (F_1,68_ = 4.3, *p* = 0.042, η^2^ = 0.067, β = −6.96, CI[−13.66, −0.25]). When women experienced/perceived greater public violence, they showed lower preferences for facial cues to high-BMI Salvadoran males (Fig. 3).

None of the other variables/factors significantly contributed to this type of preferences: participant’s age (F_1,60_ = 0.72, *p* = 0.40, η^2^ = 0.012, β = 0.45, CI[−0.62, 1.52]), access to media (F_1,60_ = 0.019, *p* = 0.89, η^2^ < 0.001, β = −0.75, CI[−11.73, 10.23]), domestic violence (F_1,60_ = 0.23, *p* = 0.88, η^2^ < 0.001, β = −0.57, CI[−8.09, 6.95]), access to health (F_1,60_ = 0.67, *p* = 0.42, η^2^ = 0.011, β = 7.14, CI[−10.36, 24.64]), illnesses (F_1,60_ = 0.28, *p* = 0.59, η^2^ = 0.005, β = 2.29, CI[−6.31, 10.90]), education (F_1,60_ = 0.35, *p* = 0.56, η^2^ = 0.006, β = 2.68, CI[−6.42, 11.79]), and having children (F_1,60_ = 0.09, *p* = 0.75, η^2^ = 0.002, β = 2.68, CI[−14.34, 19.69]).

### Discussion

Results from study 1 revealed that women who feared violence preferred cues for lower BMI in male faces. This effect was evident in Salvadoran faces but not in European faces. Hence the data support our prediction that fear of violence produces a preference for cues to low formidability in potential partners, particularly in individuals who resemble the population being studied. The effect of violence on preferences for cues of high-BMI remained significant even when controlling for participant’s sex, age, and all other factors related to health, education, and access to media. Furthermore, fearing violence (public) explained significantly more of the variation in women’s preferences for facial cues to BMI than any other factor/variable. There were no effects of fear of violence on men’s preferences.

#### The Effect of Violence Perceptions on Preferences for BMI

Facial cues to high-BMI in men have been associated with masculinity, physical strength and dominance (Holzleitner and Perrett 2016; Holzleitner et al. 2014; Snyder et al. 2011; Windhager et al. 2011). Thus, lower preferences for facial cues to men’s BMI may reflect women’s strategy to avoid those men who would be more capable of inflicting harm. In keeping with this explanation, women may be particularly disposed to evade men with high-BMI in environments where violence is high (as it is in Colombia). Since BMI has been positively correlated with masculinity (Holzleitner et al. 2014; Holzleitner and Perrett 2016), this argument follows results from Li et al. (2014) wherein women’s masculinity preferences for male faces were reduced when they were shown images of men punching women, and from Borras-Guevara et al. (2017a, b) wherein women showed lower preferences for masculine men when they had higher perceptions of public violence.

#### Stimuli Ethnicity Effect

When women perceived higher public danger from violence they showed a reduced preference for Salvadoran faces showing cues of high-BMI, but not for European faces. The effect of the ethnicity of the stimuli may be due to the physical similarity between Colombian and Salvadoran men. These two ethnic groups are both Latin American Hispanic, only separated by 1000 km, and look alike, more so than Colombians and Europeans. (For a comparison between facial averages from El Salvador, Colombia, and Europe, see Borras-Guevara et al. 2017a: Fig. 6.)

#### Effects of Different Types of Violence

In study 1 we did not find evidence that the effect of violence is most apparent for domestic rather than public violence. Perhaps this is because the survey included only one question relating to domestic violence (how much do you agree with the statement “men are dangerous to their children”?), whereas there were four questions relating to public violence. Accordingly, in study 2 we replicate the methods of study 1 but use a more extensive questionnaire with items designed to measure attitudes to domestic and public violence more fully. We also include a set of Colombian male faces.

#### Men’s Preferences for Cues to BMI

Contrary to our initial prediction, men’s preferences for facial cues to male BMI were not significantly affected by perceptions or experiences of violence. In the ANCOVA, however, men’s age did have a significant effect on facial preferences for cues to BMI of European male faces. Older men had lower preferences for European facial cues to BMI. Facial cues to BMI relate positively to physical strength and dominance (Holzleitner and Perrett 2016; Holzleitner et al. 2014; Snyder et al. 2011; Windhager et al. 2011). However, strength and muscle mass decrease with age (Keller and Engelhardt 2013; Metter et al. 1997). In fact, findings from cross-sectional and longitudinal data show a significant decline in power and upper-body strength by age 40 in men (Metter et al. 1997). Hence, our results could reflect men’s strategy to avoid high-BMI men, who would be stronger and hence more likely to win a possible agonistic encounter. As men age, they may prefer male faces that hint at more cooperative and less formidable allies.

With reference to men’s preferences for cues to high-BMI in Salvadoran male faces, the only factor that significantly contributed to explaining this type of preference was having children. Men who had children preferred Salvadoran male faces with cues to high-BMI. Previous research on life quality has revealed that being a parent decreased adults’ well-being (Simon 2008). Indeed, Evenson and Simon (2005) showed in a nationally representative sample of 10,000 adult Americans that parenthood significantly increased feelings of restlessness and fear. Our results could therefore be due to fathers feeling more vulnerable. If this were the case, father’s preferences for cues to high-BMI could reflect their need for strong allies.

Differences in BMI preferences depending on the ethnicity of the stimuli may reflect male participants’ resemblance to the Salvadoran stimuli and dissimilarity to the European stimuli. Salvadoran male faces may have been considered to represent in-group faces, prompting attraction, whereas European faces may have been perceived as part of an out-group, prompting aversion. The fact that the effects of age and having children had opposite effects on preferences for Salvadoran and European male faces may reflect just this. Physical confrontations are more likely between members of different groups, whereas cooperation is more likely between members of the same group (Van Vugt 2009). Therefore, preferring out-group male faces with cues to low-BMI would make sense when men are older and weaker. By contrast, preferring in-group male faces with cues to high-BMI could be an advantage for fathers wanting a formidable ally. We acknowledge that we had no specific hypothesis about how men’s age and fatherhood would affect BMI preferences, and our explanations are therefore post hoc.

## Study 2

### Methods

#### Participants

Recruitment methods in studies 1 and 2 were the same. However, for the second study participants were recruited in four locations: Bogota, Medellin, and suburban areas around these two cities. Although participants for study 2 were also recruited from Bogota and suburban areas, the neighborhoods and towns where recruitment occurred were different from those in study 1. A total of 236 Colombians (114 men [mean age ± SD = 31 ± 11.7] and 122 women [mean age ± SD = 34.7 ± 13.2]) were interviewed. Only data from participants who were less than 41 years old were analyzed here. This yielded a sample of 83 women (mean age ± SD = 26.7 ± 6.08) and 91 men (mean age ± SD = 26.1 ± 6.52).

#### Stimuli Used

**Fig. 4:**
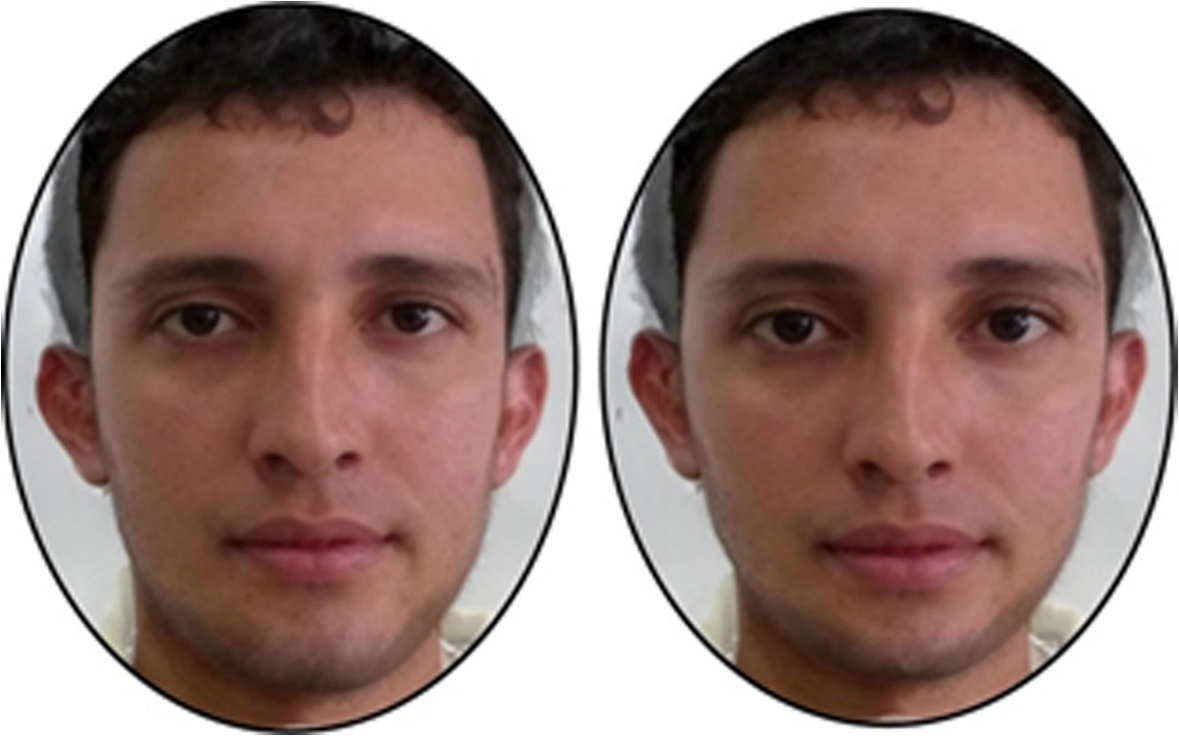
Average of five pairs of the Colombian facial stimuli. The left face corresponds to an average face exhibiting high-BMI cues and the right face low-BMI cues. (European and Salvadoran images used in this study were the same as in study 1; see Fig. 1)

Previous studies in Colombia have revealed effects of the ethnicity of facial stimuli shown to participants (Borras-Guevara et al. 2017a, b). Accordingly, three sets of face images were used in this study: European, Salvadoran, and Colombian. For details about the Salvadoran and European stimuli, please refer to study 1 and/or Batres and Perrett (2014). The Colombian photos were all taken under standard lighting/camera conditions. The subjects of the photos were instructed to pose with a neutral facial expression. All images were aligned to the same interpupillary distance and were delineated with 189 points. Five male facial composites were made by averaging/blending together the images of three Colombian men. Prototypes of facial cues to Colombian men with high-BMI and low-BMI were created. These prototypes were made by averaging the face shape of individuals with the 10 highest and lowest BMI (high-BMI M  =  27.29 kg/m^2^, SD  = 3.37; M_age_  =  24.7 years, SD  =  5.52 and low-BMI M  =  21.03 kg/m^2^, SD  =  1.40; M_age_  =  24.3 years, SD  =  4.35). These two prototypes were used as anchor points to make shape-change transforms subtracting (or adding) 50% of shape difference between the relevant composites. This resulted in five pairs of Colombian male faces (five images exhibiting facial cues of high-BMI and five of low-BMI; see Fig. 4 for an example pair).

#### Procedure

Study 2 followed procedures from study 1 but, since we included Colombian faces, participants were shown 15 pairs of faces (five European, five Salvadoran, and five Colombian) in random order, but grouped by ethnicity; hence participants had to perform 15 forced choices.

Immediately after the forced-choice task, participants answered a survey similar to the one used in study 1 but with additional questions for violence outside the home and within the family household. For violence outside the home, participants answered eight questions relating to how much they worried (on a scale from 1 to 7) about falling victim to public crimes (e.g., being attacked by a stranger, being robbed or mugged in the street, being pickpocketed, being harassed, threatened, or verbally abused in the street). Refer to the appendix for a complete list of the questions. Answers to these eight questions were averaged for subsequent analysis (Snyder et al. 2011). Participants were also asked how much in danger from violence they felt in the city/town, and how worried (on a scale from 1 to 7) they would be if they were already in bed and remembered they had left their outside door unlocked. With reference to domestic violence, participants answered seven questions (see appendix). Questions relating to violence against women were averaged together to create a new variable called “domestic violence against women.” The same was done for questions inquiring about violence against men. To avoid disclosure of personal experiences, all questions relating to violence were asked in the abstract, about the general population and not about a participant’s individual experiences. For this study, participant’s education was assessed by asking the highest level they had achieved. Eight options were given, ranging from being illiterate to having completed post-graduate studies. Participant’s access to media was determined by asking how much time they spent watching national TV, cable TV, and how frequently they used the Internet. As to participants’ health, three questions were asked: how frequently they were ill during their childhood, how many times on average they had been ill over the past year, and how they would rate their health. At the end of the experiment all participants were debriefed, paid for their time, and informed of local authorites (police and church) they could contact in case they wanted to report an incident the questionnaire had brought to mind.

#### Variables Included in the Statistical Analysis

Percentage preferences for facial cues to high-BMI were calculated for the three ethnicities in our stimuli: Colombian, European, and Salvadoran.

#### Independent Variables

Since there were several questions for each indicator (violence, education, access to media and health), when there was sufficient variability in the responses, factor analyses were run for each set of questions via principal component analysis. The factorability of sets of questions was evaluated with the same criteria and procedures as in study 1. In contrast to study 1, in study 2 separate factor analyses were conducted for men and women because the factors extracted from the questions about violence were different depending on the sex of the participant.

### Factor Analyses for Women

Since previous studies (Borras-Guevara et al. 2017a, b) and study 1 revealed a differential effect on facial preferences depending on the ethnicity of the face shown to participants, we ran separate analyses for Colombian, European, and Salvadoran facial preferences. For each model, preferences for facial cues to high-BMI (for European, Colombian, or Salvadoran male faces) were included as the dependent variable. Having children was included as a fixed factor. Age and all other variables/factors (public violence, domestic violence, TV watching, Internet use, education level, and illnesses) were included as covariates in the model.

#### Violence Factors

From the seven questions relating to violence, two factors were found. The first factor, “public violence,” mostly loaded on questions relating to vulnerability to crime, feeling danger in the city/town, and worries about leaving the home door unlocked. The second factor, “domestic violence,” mostly loaded on questions relating to partnership violence and violence against children. Respectively, these two factors explained 26.5% and 23.9% of the variance in the data (see correlation matrix in Table 2).

**Table 2:** Factors extracted from the questions relating to violence (**bold** indicates correlation values >0.42 or < −0.42)

Violence	Public violence	Domestic violence
Average violence against women	0.27	**0.67**
Average violence against men	−0.06	**0.86**
Men dangerous to children	0.42	**0.56**
Average vulnerability	**0.60**	0.03
Danger city/town	**0.67**	−0.19
Danger country	**0.65**	−0.35
Locking door	**0.61**	0.40

#### Health Factors

Questions relating to participants’ illnesses (frequency of illnesses during the last year, during childhood, and health rating) were all included in a factor analysis. Only one factor explaining 45.9% of the variance was extracted. From this point forward this factor will be referred to as “illnesses.” Other questions relating to health (access to a hospital and drinking water) did not meet the Kayser-Meyer-Olkin adequacy principles and hence were not include (Table 3).

**Table 3:** Factors extracted from the questions relating to health with their corresponding loadings. Loadings in **bold** show values >0.47 or < −0.47

Health	Illnesses
Health rating	**−0.76**
Average illnesses reported for the last year	**0.47**
Frequency of illnesses during childhood	**0.76**

#### Access to Media

Two of the three questions regarding access to media (times spent watching national TV, cable TV, and frequency of Internet use) were included in a factor analysis. The only factor extracted (“TV watching”) loaded on both time spent watching national TV and time spent watching cable TV at 0.87, explaining 74% of the variance. Internet use was included as a binary variable (low and high use) for subsequent analyses.

#### Education Level

This question was introduced in subsequent analyses on its own since it was the only question asked in reference to participant’s education level.

### Factor Analyses for Men

#### Violence Factors

Three factors were extracted for questions relating to experiences and perceptions of violence. The first factor, “general violence,” loaded highly on all questions. The second, “violence type 2,” mostly loaded on questions relating to fear when leaving the outside door unlocked, average vulnerability to public crime, and domestic violence against women. The third factor, “violence type 3,” was mostly loaded by perceptions of violence in the city/town. Respectively, these three factors explained 30.9%, 18.10%, and 15.03% of the variance (see correlation matrix in Table 4). Labeling factors 2 and 3 was not straightforward because of the tight relationship between answers to very different questions relating to different types of violence.

**Table 4:** Factors extracted from the questions relating to violence (**bold** indicates correlation values >0.46 or < −0.46)

Violence	General violence	Violence type 2	Violence type 3
Average violence against women	**0.65**	**0.50**	−0.13
Average violence against men	**0.71**	0.24	−0.30
Men dangerous to children	**0.46**	0.33	−0.23
Average vulnerability	**0.59**	**−0.63**	0.05
Danger city/town	0.19	0.21	**0.82**
Danger country	**0.54**	0.09	**0.46**
Locking door	**0.59**	**−0.62**	−0.06

#### Health Factors

Two factors were extracted from the questions relating to men’s health, explaining 39.6% and 36.1% of the variance, respectively. The first factor mostly loaded with questions relating to illnesses, and the second factor related mostly to current health rating (Table 5).

**Table 5:** Factors extracted from the questions relating to health with their corresponding loadings. Loading in **bold** show values >0.33 or < −0.33

Health	Illnesses	Health
Health rating	−0.33	**0.83**
Average illnesses reported for the last year	**0.62**	**0.61**
Frequency of illnesses during childhood	**0.83**	−0.12

#### Access to Media

A single factor was extracted from the two questions included in this analysis: time spent watching national TV and time spent watching cable TV, explaining 68.8% of the variance. Internet use was included as a binary variable (low and high use) for subsequent analyses.

#### Education Level

Again this question was introduced in subsequent analyses on its own.

### Results

#### Women’s Preferences for High-BMI European Males

The only two factors that significantly affected preferences for facial cues to high-BMI European males were TV watching (F_1,69_ = 45.87, *p* = 0.018, η^2^ = 0.078, β = 7.12, CI[1.26, 13.08]) and illnesses (F_1,69_ = 4.48, *p* = 0.038, η^2^ = 0.061, β = 6.55, CI[0.37, 12.73]). Women who spent more time watching TV and who reported having poorer health (more illnesses) had a higher preference for facial correlates of high-BMI European males. No other variables/factors contributed to explaining facial preferences for cues to BMI: having children (F_1,69_ = 0.08, *p* = 0.68, η^2^ = 0.002, β = −1.96, CI[−15.27, 11.34]), public violence (F_1,69_ = 0.005, *p* = 0.94, η^2^ < 0.001, β = 2.09, CI[−5.79, 6.21]), domestic violence (F_1,69_ = 0.10, *p* = 0.77, η^2^ = 0.001, β = 1.03, CI[−5.39, 7.45]), Internet use (F_1,69_ = 0.20, *p* = 0.65, η^2^ = 0.003, β = 3.67, CI[−12.57, 19.91]), and education (F_1,69_ = 0.49, *p* = 0.48, η^2^ = 0.007, β = 1.97, CI[−3.65, 7.60]).

#### Women’s Preferences for High-BMI Salvadoran Males

**Fig. 5:**
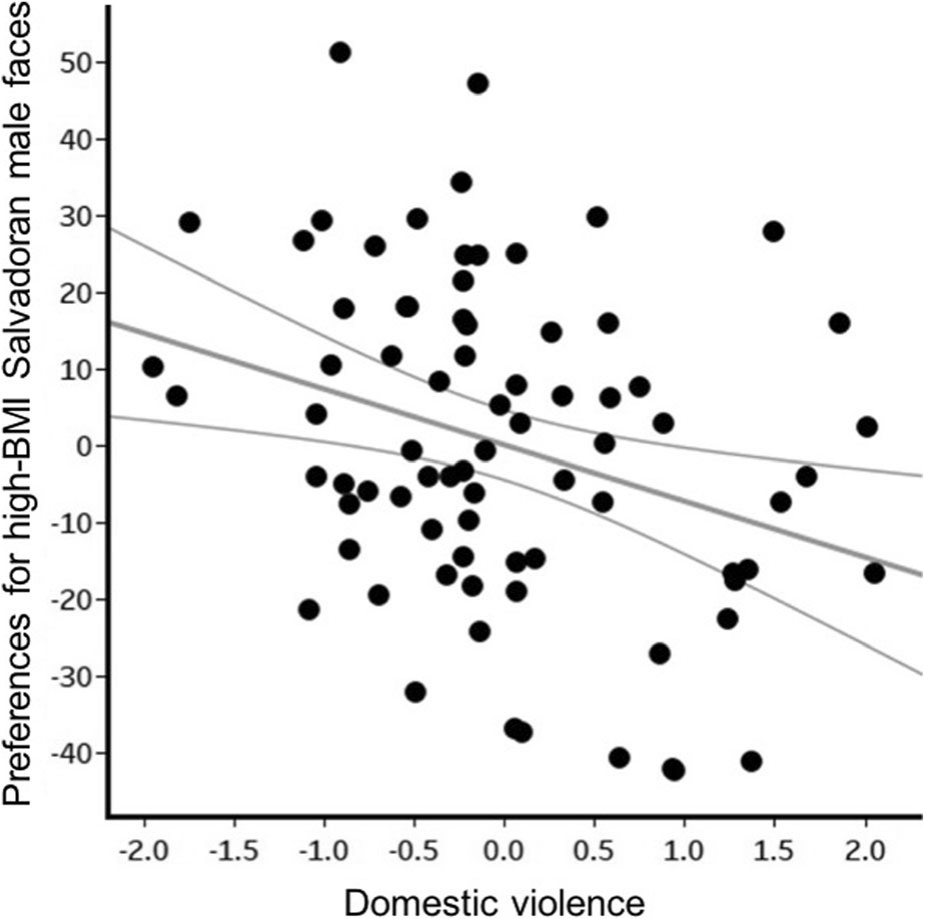
Effect of violence within partnership on women’s preferences for facial cues to high BMI males. Participants are represented by black dots in the graph. Grey lines show a 95% confidence interval. (Residual domestic violence plotted against the unstandardized residuals of preferences for high BMI, controlling for age, frequency of Internet use, education, illnesses, public violence and having children)

Preferences for facial cues for high-BMI among Salvadoran males were affected by domestic violence (F_1,69_ = 7.06, *p* = 0.010, η^2^ = 0.093, β = −7.30, CI[−12.78, −1.82]). Women who had higher perceptions of risk of partnership violence showed lower preferences for cues to high-BMI among Salvadoran males (Fig. 5). Additionally, women who spent more time watching TV had a lower preference for such men than women who watched less TV (F_1,69_ = 5.89, *p* = 0.018, η^2^ = 0.079, β = −6.13, CI[−11.17, −1.09]). No other factor/variable significantly predicted women’s preferences for facial cues to high-BMI (age: F_1,69_ = 0.016, *p* = 0.90, η^2^ < 0.001, β = 0.06, CI[−0.89, 1.09]; Internet use: F_1,69_ = 0.92, *p* = 0.34, η^2^ = 0.013, β = −6.66, CI[−20.52, 7.20]; education F_1,69_ = 0.80, *p* = 0.37, η^2^ = 0.011, β = −2.15, CI[−6.96, 2.65]; illnesses: F_1,69_ = 0.03, *p* = 0.87, η^2^ < 0.001, β = 0.43, CI[−4.84, 5.69]; public violence: F_1,69_ = 1.66, *p* = 0.20, η^2^ = 0.024, β = 3.31, CI[−1.81, 8.44]; and having children: F_1,69_ = 3.52, *p* = 0.065, η^2^ = 0.049, β = −10.68, CI[−22.04, 0.67]).

#### Women’s Preferences for High-BMI Colombian Males

Preferences for facial cues for high-BMI among Colombian males were not affected by domestic violence (F_1,69_ = 0.35, *p* = 0.55, η^2^ = 0.005, β = −1.90, CI[−8.31, 4.50]) or by any other variable/factor: age (F_1,69_ = 0.65, *p* = 0.42, η^2^ = 0.009, β = 0.45, CI[−0.66, 1.56]), Internet use (F_1,69_ = 1.45, *p* = 0.23, η^2^ = 0.021, β = −9.78, CI[−25.98, 6.42]), education (F_1,69_ = 0.43, *p* = 0.51, η^2^ = 0.006, β = −1.84, CI[−7.46, 3.77]), TV watching (F_1,69_ = 0.00, *p* = 0.99, η^2^ < 0.001, β = −0.02, CI[−5.92, 5.87]), illnesses (F_1,69_ = 0.13, *p* = 0.72, η^2^ = 0.002, β = −1.11, CI[−7.27, 5.05]), public violence (F_1,69_ = 2.39, *p* = 0.13, η^2^ = 0.034, β = 4.64, CI[−1.34, 10.63]), or having children (F_1,69_ = 1.04, *p* = 0.31, η^2^ = 0.015, β = 6.78, CI[−6.49, 20.05]).

#### Men’s Preferences for High-BMI European Males

None of the covariates included in the model significantly affected preferences for facial cues to high-BMI among European males: having children (F_1,81_ = 0.38, *p* = 0.54, η^2^ = 0.005, β = −5.33, CI[−22.64, 11.96]), general violence (F_1,81_ = 0.38, *p* = 0.54, η^2^ = 0.005, β = −5.33, CI[−22.64, 11.96]), violence type 2 (F_1,81_ = 0.38, *p* = 0.54, η^2^ = 0.005, β = −5.33, CI[−22.64, 11.96]), violence type 3 (F_1,81_ = 0.38, *p* = 0.54, η^2^ = 0.005, β = −5.33, CI[−22.64, 11.96]), Internet use (F_1,81_ = 0.079, *p* = 0.78, η^2^ = 0.001, β = −1.79, CI[−14.53, 10.94), education (F_1,81_ = 1.9, *p* = 0.18, η^2^ = 0.025, β = 1.38, CI[−3.34, 6.11), TV watching (F_1,81_ = 0.13, *p* = 0.71, η^2^ = 0.002, β = 1.15, CI[−5.1, 7.40]), illnesses (F_1,81_ = 0.73, *p* = 0.39, η^2^ = 0.019, β = 2.62, CI[−3.49, 8.75]), health (F_1,81_ = 0.58, *p* = 0.45, η^2^ = 0.008, β = −2.45, CI[−8.78, 3.93]) or age (F_1,81_ = 0.61, *p* = 0.44, η^2^ = 0.009, β = 0.44, CI[−0.69, 1.59]).

#### Men’s Preferences for High-BMI Salvadoran Males

There was a trend for significance for the effect of TV watching (F_1,81_ = 3.38, *p* = 0.07, η^2^ = 0.046, β = 4.89, CI[−0.41, 10.18]) on men’s preferences for facial traits related to high-BMI Salvadoran men. Men who watched TV for a longer time had higher preferences for high-BMI faces. None of the other covariates included in the model significantly affected this type of preference: having children (F_1,81_ = 0.23, *p* = 0.63, η^2^ = 0.003, β = 3.66, CI[−11.29, 16.62]), general violence (F_1,81_ = 2.19, *p* = 0.14, η^2^ = 0.030, β = 3.74, CI[−1.29, 8.77]), violence type 2 (F_1,81_ = 0.06, *p* = 0.80, η^2^ = 0.001, β = 0.66, CI[−4.61, 5.95]), violence type 3 (F_1,81_ = 0.43, *p* = 0.51, η^2^ = 0.006, β = −1.62, CI[−6.51, 3.27]), Internet use (F_1,81_ = 0.07, *p* = 0.78, η^2^ = 0.001, β = −1.79, CI[−14.53, 10.94]), education (F_1,81_ = 0.34, *p* = 0.56, η^2^ = 0.005, β = 1.38, CI[−3.35, 6.11]), illnesses (F_1,81_ = 1.30, *p* = 0.26, η^2^ = 0.018, β = −2.97, CI[−8.17, 2.21]), health (F_1,81_ = 0.54, *p* = 0.46, η^2^ = 0.008, β = −1.99, CI[−7.37, 3.38]) or age (F_1,81_ = 0.53, *p* = 0.47, η^2^ = 0.008, β = 0.36, CI[−0.62, 1.34]).

#### Men’s Preferences for High-BMI Colombian Males

There was a trend for significant effects of education (F_1,81_ = 3.5, *p* = 0.06, η^2^ = 0.047, β = −5.37, CI[−11.09, 0.35]). More-educated men had lower preferences for high-BMI Colombian male faces. None of the other covariates contributed to explaining these preferences: having children (F_1,81_ = 1.24, *p* = 0.27, η^2^ = 0.017, β = −9.99, CI[−27.90, 7.92]), general violence (F_1,81_ = 0.001, *p* = 0.98, η^2^ < 0.001, β = 0.078, CI[−6.06, 6.21]), violence type 2 (F_1,81_ = 0.94, *p* = 0.33, η^2^ = 0.013, β = −3.12, CI[−9.54, 3.29]), violence type 3 (F_1,81_ = 0.13, *p* = 0.71, η^2^ = 0.002, β = 1.10, CI[−4.88, 7.08]), Internet use (F_1,81_ = 0.50, *p* = 0.48, η^2^ = 0.007, β = −5.46, CI[−20.79, 9.87]), illnesses (F_1,81_ = 0.053, *p* = 0.82, η^2^ = 0.001, β = 0.73, CI[−5.60, 7.07]), health (F_1,81_ = 0.02, *p* = 0.89, η^2^ < 0.001, β = −0.47, CI[−7.05, 6.10]) or age (F_1,81_ = 0.013, *p* = 0.91, η^2^ < 0.001, β = 0.067, CI[−1.11, 1.25]).

## General Discussion

Prior studies indicated that perceptions of violence have a negative impact on women’s preferences for male facial masculinity. Here two studies extend those findings and reveal that violence also negatively impacts preferences for apparent BMI. Both high-BMI and high masculinity are attributes that indicate a man is more dangerous and formidable. The effect of violence on BMI preferences was apparent in both studies 1 and 2, lowering preferences for male faces indicative of high-BMI. Although the influence of violence was evident in analyzing the preferences of both men and women, the effects were mostly driven by women’s preferences. Additionally, violence effects were sensitive not only to the type of violence but also to the ethnicity of the face shown to participants. We note that our measure of preference could reflect attraction to a potential spouse, a neighbor, or a friend. Nonetheless many of the benefits from an opposite-sex friend may overlap with the benefits from a partner (Bleske-Rechek and Buss 2001).

### Effects of Different Types of Violence

In study 2, women who felt they were at higher risks of partnership violence (domestic violence) had significantly lower facial preferences for cues to high-BMI among Salvadoran males. This result aligns with previous findings that women who worry more about domestic violence have lower preferences for formidable men in terms of facial masculinity (Borras-Guevara et al. 2017b). We did not, however, find any effects of public violence on BMI preferences, unlike our findings in study 1. Differences in effects of domestic violence between the two studies may reflect modifications in the questions participants were asked. In study 1, people were asked to evaluate the statement “men are dangerous to their children.” In study 2, domestic violence was assessed through six additional questions. The inclusion of questions that were more specific and relevant to violence within the household in study 2 may have improved the chances of detecting negative effects of domestic violence on preferences for formidable (high-BMI) men.

Differences between the results from studies 1 and 2 may reflect that the effects of domestic violence trump those of public violence. The influence of domestic violence on partner preferences appears stronger than that of public violence for both masculinity (Borras-Guevara et al. 2017b) and BMI.

### Effects of Stimulus Ethnicity

The effect of public violence on facial preferences for cues to men’s BMI was evident for Salvadoran male faces, but not for European faces. In study 1, participants with higher perceptions of danger from public violence showed a significantly lower preference for facial cues of high-BMI among Salvadoran males. Furthermore, women in study 2 also displayed lower preferences to high-BMI Salvadoran male faces when they worried more about domestic violence. Stimulus ethnicity effects in study 1 may be due to Colombian women being more exposed on a daily basis to the risks associated with interacting with Colombian men who look more like the Salvadoran than the European stimuli.

Although this explanation may account for the results in study 1, it does not account for the absence of effects for the Colombian stimuli in study 2. The absence of effects for Colombian faces might reflect that high-BMI can result from increased proportions of muscle and/or fat. Furthermore, the extent to which the effects of BMI are evident in the face may differ depending on the sample population (Batres et al. 2017). Hence, the manufacture of stimuli may in a given sample relate more to muscularity and in a different sample may relate more to adiposity. For the stimuli used here, the Salvadoran and Colombian men may portray different amounts of fat and muscle. It may be the case that the high-BMI Salvadoran stimuli indicate a higher muscle composition than that of the high-BMI Colombian stimuli, making the Salvadoran stimuli look more dangerous than the Colombian stimuli. Therefore women who worry about domestic violence should fear the high-BMI Salvadoran stimuli more than the high-BMI Colombian stimuli. (See Figs. 1 and 4 for a comparison between the average faces of high and low-BMI cues of Salvadoran and Colombian males.)

Regardless of the explanation for the variation in effects of ethnicity of facial stimuli, the research reported here parallels previous reports in demonstrating that perceived level of violence is an important consideration for preferences including those relevant to mate choice (Borras-Guevara et al. 2017a, b; Snyder et al. 2011). Further research should focus in isolating the influence of facial cues to muscle and fat in shaping preferences (Holzleitner et al. 2014; Phalane et al. 2017).

### Direction of Violence Effects

To our knowledge, previous research has not investigated the effects of experienced or perceived violence on preferences for facial correlates of BMI. Instead most research has focused on masculinity preferences. Since facial masculinity has been positively correlated with facial cues of BMI (Holzleitner et al. 2014; Holzleitner and Perrett 2016), we expected that preferences for facial cues of BMI would follow the same pattern of masculinity preferences. In the past, when violence has been taken into account, masculinity preferences have been explained in terms of women wanting protection from their partners. For example, Brooks et al. (2010) noted that higher homicide rates predicted higher masculinity preferences across 30 countries and Snyder et al. (2011) found that women who felt more vulnerable to crime preferred partners who were described as formidable. Nonetheless, Borras-Guevara et al. (2017a, b) recently suggested that women’s lower masculinity preferences when fearing more about domestic and public violence, may be a strategy to avoid males who are stronger, more aggressive and formidable, who could be more capable of harming women. Our results here show the same pattern, women preferring lower facial correlates of BMI when they have higher perceptions of public and domestic violence. These results may reflect that indeed men’s facial correlates of BMI and masculinity are cues to formidability and strength, which women may want to avoid in a romantic relationship context.

The difference between our results and those of Brooks et al. (2010) may be due to their analysis being at the population level whereas ours was at the individual level (Pollet et al. 2014). One of the drawbacks of running analyses at the population level is that any effect may reflect the influence of one of several interrelated factors. For example income inequality is associated to violence across countries, while also correlating with indices of poor health. Broad measures of health at the country level, homicide and income equality may reflect only a small portion of the actual influence of health and violence on individuals.

### Effect of Illnesses

Study 2 (but not study 1) revealed an effect of the illnesses factor. Women who reported more recent illnesses and illness during their childhood, expressed higher preferences for facial cues to high-BMI European males. To the extent that men’s high-BMI and masculinity have similar good health connotations, this effect parallels the findings of De Barra et al. (2013), where higher childhood illness in a Bangladesh population predicted increased female preferences for male masculinity (though see Scott et al. 2013). Increased BMI (while remaining below an obese weight) may show resistance to disease (Phalane et al. 2017).

### Effect of Media Access

Women’s access to media (in study 2: TV watching) significantly influenced preferences for cues to high-BMI, for both Salvadoran and European male faces. When women spent more time watching TV (national and cable), they preferred European male faces showing cues of high-BMI, but low-BMI Salvadoran male faces. The discrepancy between the effects of TV watching on preferences for cues to BMI found for Salvadoran and European male faces may be due to the fact that non-white men (blacks and latinos) are usually portrayed in TV as more violent than white men. Dixon and Linz (2000) reported significant racial profiling in television news in the United States; Latino men were portrayed as lawbreakers and white men as law defenders. Colombian women who watch cable TV have access to American TV channels, hence their preference may reflect the racial biases portrayed by the media. The fact that no significant effects were found for the Colombian stimuli may reflect a different body composition (muscle vs. fat) between the Colombian and Salvadoran stimuli as discussed above.

### Effects of Violence Depending on Participant’s Sex

Across the two studies presented here, we consistently find that violence affects women’s BMI preferences for male faces. By contrast, effects of social indicators on men’s preferences for facial cues to BMI are inconsistent between studies 1 and 2. This discrepancy between effects for women and men may reflect that violence is in fact relevant for women’s mate choice. The nature of the question asked to participants (which face do you consider most attractive?) may have also contributed to finding violence effects only in women. We initially predicted that effects of violence on men’s preferences for cues to BMI would parallel those of women as men would be aware of what women want or that men’s preferences would reflect the pursuit of cooperative allies. It is possible that more specific questions (which of the two faces, would be preferred by women? or which face would you prefer as an ally?) would have confirmed (or contradict) this prediction more efficiently. Future experiments should take this into account when studying men’s preferences for male faces more thoroughly.

## Conclusion

Previous research has pointed out the trade-off faced when preferring certain male characteristics: large, strong, masculine men are of high value in some contexts (e.g., intrasexual competition) while being at a disadvantage and representing danger in others (Borras-Guevara et al. 2017a, b; Li et al. 2014; Snyder et al. 2011). In line with the costs of this trade-off we report a negative relationship between women’s preferences for facial cues to men’s BMI and perceptions of danger from public and domestic violence. The effect of violence on face preferences was apparent in faces that were ethnically similar to the participants. Furthermore, the effect of violence remained significant after controlling for other factors known to affect preferences (e.g., age, education, access to media, health, and having children). Women’s preferences for men’s facial cues to BMI, follow the pattern of preferences of male facial masculinity to some extent. Traits related to high masculinity and high-BMI may both afford protection and constitute danger. Levels of public and domestic violence will determine the relative costs and benefits of formidable men. While previous research has focused on the effect of environmental harshness (e.g., access to development, access to media) our findings point out that women’s mate preferences are also affected by perceptions of violence.

### Electronic supplementary material

ESM 1 (XLSX 34 kb)

## Appendix

### Questionnaire for study 1 (English version)

1. **Demography**
  1. What is your gender?
  2. What is your sexual orientation? (1–7, 1 = completely homosexual, 4 = bisexual, and 7 = completely heterosexual)
  3. Where are you from?
  4. How old are you?
  5. Do you have any children? (Yes/No)
2. **Education**
  1. Did you attend high-school? (Yes/No)
  2. Did you graduate from high-school? (Yes/No)
  3. Did you attend university? (Yes/No)
3. **Development**
  1. Do you have electricity at home? (Yes/No)
  2. Do you have television at home? (Yes/No)
  3. Do you have Internet at home? (Yes/No)
  4. How many times a year do you have access to the Internet? (1 = every day, 2 = every week, 3 = every month, 4 = every 3 months, 5 = every 6 months, 6 = once a year, 7 = never)
4. **Health**
  1. Do you have easy access to a hospital? (Yes/No)
  2. On average, how many times a year do you get sick?
  3. Do you have potable water at home? (Yes/No)
  4. Were you born at a hospital?
  5. During your childhood (before age 13) how frequently did you get a serious illness that confined you to bed?
5. **Violence perceptions and experiences**
  1. In general how much in danger from violence do you feel in the following places? (1–7, with 1 being not at all in danger and 7 being very much in danger).
    - At home
    - In the city/town
    - In the country
  2. Over the last year, how frequently have you or anyone you know been victim of an actual or attempted mugging or physical attack?
  3. How much do you agree with the following statement? **“Men are dangerous to their own children”** (1–4, 1 = not at all, 2 = somewhat disagree, 3 = somewhat agree, 4 = fully agree).

### Questionnaire for study 2 (English version)

1. **Demography**
  1. How old are you?
  2. Please indicate your gender at birth.
    - a. Female
    - b. Male
    - c. Other
    - d. Prefer not to say
      i. If female, at what age did you get your first period?
  3. Please indicate how would you rate your sexual orientation? (1–7, 1 = completely homosexual, 4 = bisexual, and 7 = completely heterosexual) You can skip this question if you would prefer.
  4. Are you currently in a committed relationship? (Yes/No)
    - a. If not, would you like to be in a committed relationship? (Yes/No)
  5. Do you have any children? (Yes/No)
    - a. If yes, how many children do you have?
    - b.If yes, please indicate what is the age of your youngest child: ____
2. **Health**
  1. In general, how would you rate your health? (1 = poor – 7 = excellent)
  2. On average how many times a year do you get sick, so that you are confined to stay in bed?
  3. During childhood (before 13 years old) how often in a year did you get a serious illness that confined you to bed? (1 = never – 7 = more than 5 times a year)
3. **Education**
  1. Please indicate the highest level of education you attained or are currently enrolled in: illiterate, attended primary school, completed primary school, attended secondary school, completed secondary school, attended university, completed undergraduate degree, attended postgraduate degree, completed postgraduate degree.
4. **Development**
  1. How many times a week do you have access to the Internet?
    - a.0–2
    - b.2–4
    - c.4–6
    - d.All the time
  2. Do you have TV at home? (Yes/No)
    - a.If you answered yes, how much national TV you watch per day?
      i. 30 minutes
      ii. 1 hour
      iii. 2 hours
      iv. 3 hours
      v. More than 3 hours
  3. Do you have foreign/cable TV at home?
    - a.If you answered yes, how much foreign TV you watch per day?
      i. 30 minutes
      ii. 1 hour
      iii. 2 hours
      iv. 3 hours
      v. More than 3 hours
5. **Fear of violence**
  1. In general, how much in danger from violence do you feel in the following places? (1 = “feel no danger at all” – 7 = “feel very much in danger”).
    - a.In town/city
    - b.In the country
  2. How worried do you feel when you are already in bed and you realize you have forgotten to lock/block your home front door? (1 = “not at all worried, I would sleep like a baby” – 7 = “very worried, I would jump out of bed straight away to lock/block the door”).
6. **Perceived vulnerability to public violence**

How much do you worry about falling victim of the following crimes on a regular basis? (1 = not at all – 7 = all the time)

1. Being attacked by a stranger in the street
2. Being robbed or mugged in the street
3. Being harassed, threatened or verbally abused in the street
4. Being pickpocketed
5. Having something stolen in a violent manner
6. Having your home or property vandalized
7. Having someone break into your home whilst you or your family are there
8. Having someone break into your home whilst the inhabitants are away

7. **Domestic violence**
  1. How likely is a woman to be the target of domestic/partner violence in your area (e.g., neighborhood, town)? (1 = not likely at all – 7 = very likely)
  2. How likely is a guy to be the target of domestic/partner violence in your area (e.g., neighborhood, town)? (1 = not likely at all – 7 = very likely)
  3. How much do you agree with the following statement “Men are dangerous to their own children” (1 = completely disagree – 7 = completely agree).
8. **Vulnerability within own relationship**
  1. How vulnerable do women (in your area/ town) feel if they have a confrontation with their partner? (1 = not vulnerable at all – 7 = very vulnerable).
  2. If a woman (in your town) disagrees with her partner about something that really matters to her, is she likely to feel safe enough to tell him (1 = very likely to feel safe enough – 7 = not very likely to feel safe enough).
  3. How vulnerable do men (in your area/ town) feel if they have a confrontation with their partner? (1 = not vulnerable at all – 7 = very vulnerable).
  4. If a man (in your town) disagrees with his partner about something that really matters to him, is he likely to feel safe enough to tell her (1 = very likely to feel safe enough – 7 = not very likely to feel safe enough).
